# Should iterative spinal surgeries be performed? A case report

**DOI:** 10.1093/jscr/rjac304

**Published:** 2022-07-05

**Authors:** George Ampat, Samantha J Rhodes, Jonathan MG Sims, Emily Wyman

**Affiliations:** School of Medicine, Institute of Life Course & Medical Sciences, Faculty of Health & Life Sciences, University of Liverpool, Liverpool, UK; Research Unit, Talita Cumi Ltd., Southport, UK; Research Unit, Talita Cumi Ltd., Southport, UK; School of Medicine, Institute of Life Course & Medical Sciences, Faculty of Health & Life Sciences, University of Liverpool, Liverpool, UK

## Abstract

The number of spinal surgeries performed is increasing. Along with this comes an increase in iterative surgeries. Each surgery that a patient undergoes has an increased risk of complication and a decreased success rate compared to the last. We present a case of a 51-year-old female who continues to experience debilitating low back pain following three double fusions performed over four years. The patient describes that she is in more pain now than before any previous surgical intervention. Following these surgeries, the patient has been forced to take early retirement, frequently uses a wheelchair and requires assistance with daily activities. The role of iterative surgery in healthcare needs to be re-assessed. The poor outcomes, especially following tertiary and quaternary surgery, question the use of iterative surgery entirely.

## INTRODUCTION

The number of spinal surgeries performed is increasing. A study looking at lumbar fusion procedures in the USA found a 62.3% increase in the number performed between 2004 and 2015 [1]. This increase has also led to a rise in iterative surgeries. Two years following spinal surgery, 12.5% of patients underwent a repeat surgery, which increased to 19.3% four years following the surgery [2]. Other studies have found reportedly higher iterative surgery rates, with Howe *et al.* finding 35% of patients underwent a secondary surgery at a mean follow-up of two years [3].

The complications associated with spinal surgery can be serious and debilitating for the patient. The rate of complications for spinal fusion surgery has been reported to be as high as 52.2%. However, this includes minor complications, too [4]. The risk of minor and severe complications increases with each iterative surgery. Though a repeat surgery intends to improve the outcome obtained from the earlier intervention, studies show that poorer outcomes result with each further surgery. A review from 2012 suggested that only 30% of secondary surgeries are successful [5]. Furthermore, the succession rate decreased, with tertiary and quaternary surgeries being successful 15 and 5% of the time, respectively [5].

On average, iterative surgeries include secondary and tertiary surgery, but extreme cases have also been reported. A case report on iterative surgeries for a single patient enumerated 27 surgeries within the space of two years [6]. This is an extreme case, but it highlights the importance of reporting cases of iterative surgery. There seems to be a paucity of information on iterative surgery, which only reinforces the need to report them to improve patient outcomes.

## CASE REPORT

A 51-year-old female presented elsewhere in March 2014 with lower back pain. The patient had suffered from chronic low back pain for several years prior and had also begun to experience severe groin pain and left-sided posterior hip pain in the months preceding. The patient had taken time off work because of her symptoms but had not participated in a pain management or functional rehabilitation programme. Previous treatment had only included pain medications prescribed by the GP.

**Figure 1:**
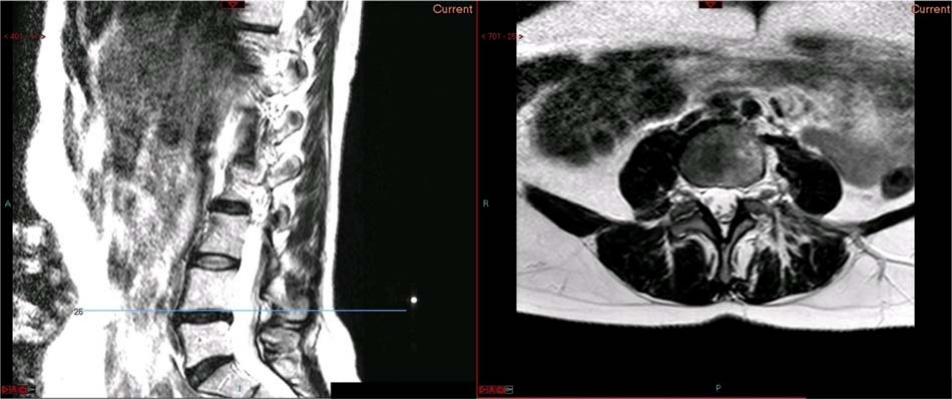
Preoperative MRI scan of the lumbar spine: preoperative MRI scan of the lumbar spine performed in October 2013, showing non-compressive disc degeneration at the L4/5 and L5/S1 vertebrae.

**Figure 2:**
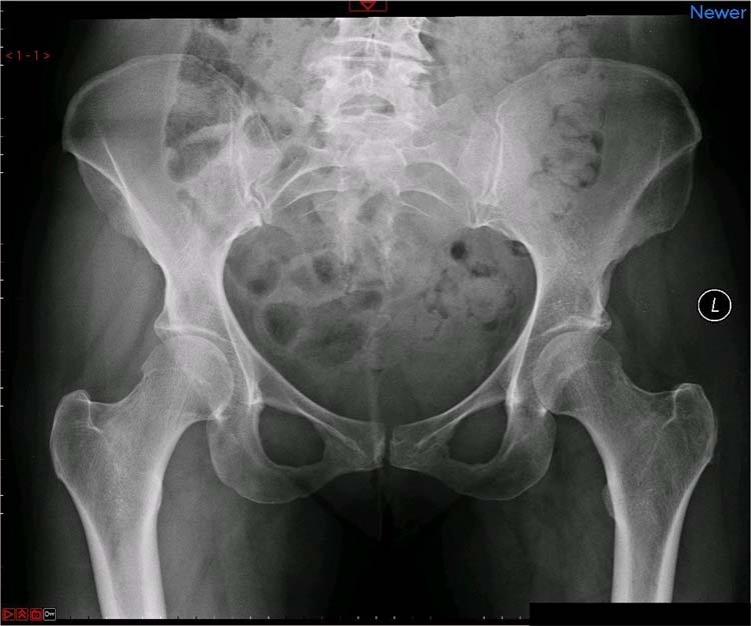
Preoperative X-ray of the pelvis: preoperative X-ray of the pelvis, performed in March 2014, showing no implants yet in place.

**Figure 3:**
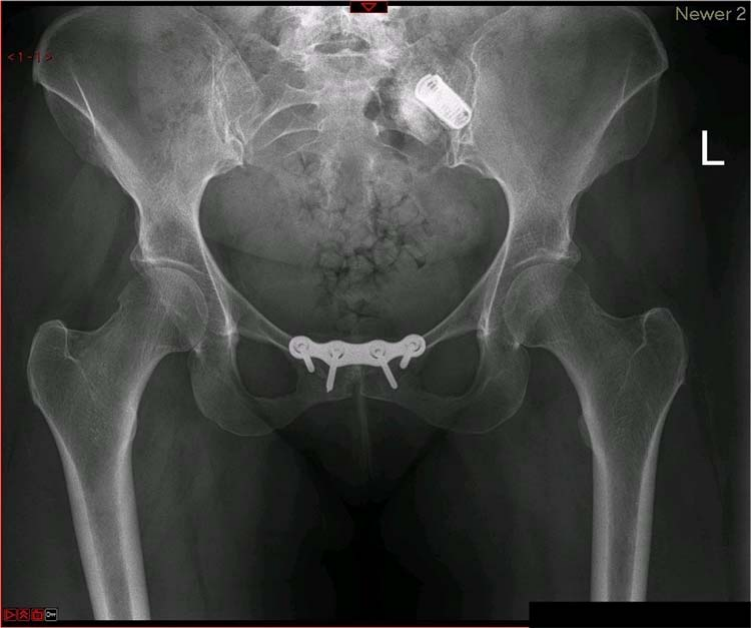
Postoperative X-ray of the pelvis following the first fusion surgery: postoperative X-ray of the pelvis, performed in November 2014 following the first fusion surgery, showing plating of the symphysis pubis and distraction arthrodesis of the left sacroiliac joint with DIANA implant.

**Figure 4:**
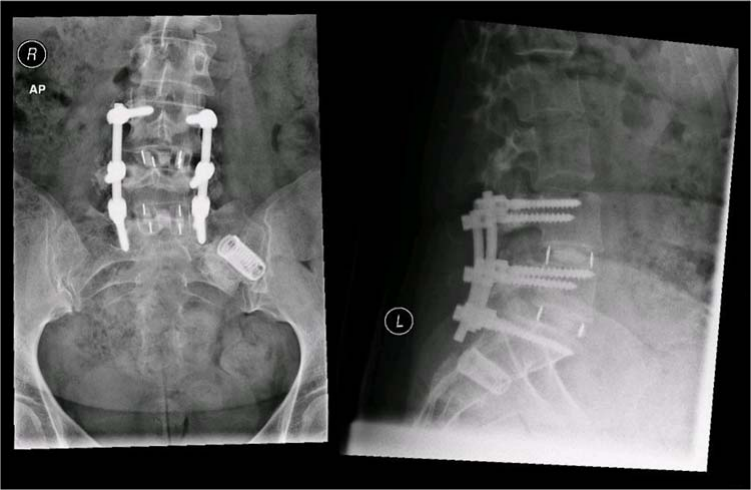
Postoperative X-ray of the pelvis following the second fusion surgery: postoperative X-ray of the pelvis, performed in January 2016 following the second fusion surgery, showing 360° (front and back) fusion of the L4/5 and L5/S1 disc space.

**Figure 5:**
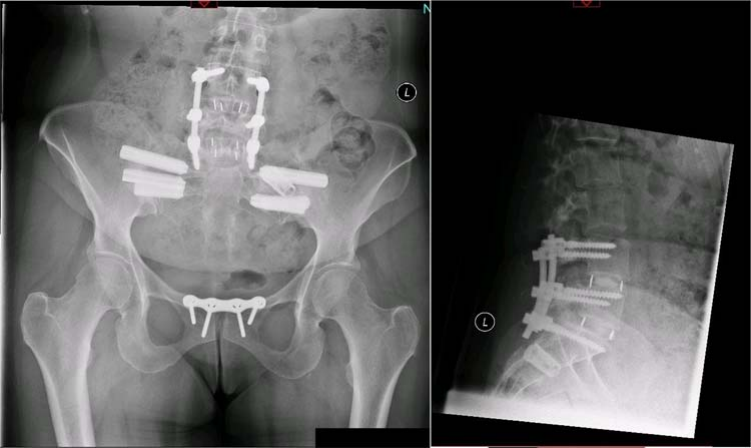
Postoperative X-ray of the pelvis following the third fusion surgery: postoperative X-ray of the pelvis, performed in March 2021 following the third fusion surgery, showing refusion of the left sacroiliac joint and primary fusion of the right sacroiliac joint with the SI-bone implant.

A magnetic resonance imaging (MRI) scan performed in October 2013 (Fig. 1) identified non-compressive, degenerated discs at L4/5 and L5/S1 vertebral levels. However, clinically as the patient was more tender over the left SI joint and the pubic symphysis (Fig. 2), a left sacroiliac joint fusion and symphysis pubis plating procedure was performed in August 2014 (Fig. 3). Following a short period of relief, the pain returned. Subsequently, the patient underwent a 360° fusion (front and back) of the L4/L5 and L5/S1 discs in August 2015 (Fig. 4), a year following the previous surgery. Once again, following a brief period of relief, the symptoms returned. The assumption then was that the initial fusion of the left sacroiliac joint had failed. Therefore, a revision fusion of the left sacroiliac joint and a primary fusion of the right sacroiliac joint was undertaken in August 2017 (Fig. 5).

Despite undergoing three double spinal fusion operations, the patient continues to experience debilitating pain. The patient describes the pain as worse now than before she underwent any spinal surgical procedure. The patient is unable to carry out daily activities and uses a wheelchair for a significant amount of her daily life.

To try and mitigate her current pain, the patient presently takes the following medications:

- Pregabalin 200 mg—thrice a day.
- Zomorph 10 mg—twice a day
- Oramorph 5–10 ml—every 3–4 h if she needs it.
- Diclofenac suppositories
- Diazepam—only when the pain is extremely severe.

## DISCUSSION

The number of spinal surgeries that are performed has increased dramatically over the last two decades [1]. Along with this, the number of iterative surgeries has increased. Iterative surgery is associated with a high complication rate and a low success rate [5]. It is essential to report the outcomes of iterative surgeries to highlight the significant issues and improve patient outcomes. This case report presents a patient who underwent a total of three double fusions and is now in more pain. This, therefore, raises the question, would the patient have been better off not having any of the three surgeries?

The rate at which iterative surgeries occur is mixed throughout the literature. Reports have varied between one in every five to one in every three patients undergoing at least a secondary surgery [2, 3]. Different inclusion criteria between studies can be partly attributed to this variation. However, a study that evaluated the incidence of iterative lumbar surgeries in 29 529 patients found differences between hospitals and between surgeons [7]. This shows the uncertainty and the lack of clear guidelines regarding the indications for these procedures.

The outcomes decrease with each further intervention. A review from 2012 stated that with second, third and fourth surgeries, no >30, 15 and 5% result in success, respectively [5]. Albert Einstein famously defined insanity as doing the same thing over and over again and expecting different results. Iterative surgery should hold the same principle. When the first surgery fails, and even more so when the second surgery fails, would it be insanity to continue to attempt to fix the same problem?

There is much uncertainty surrounding the use of iterative surgeries. The criteria used to distinguish the need for iterative surgery differs between hospitals and surgeons. In addition, there is a low success rate. With each iterative surgery, outcomes decrease consecutively. There is a need for the development of standardized criteria for iterative surgeries. The patient should be clearly informed of the diminishing returns from iterative surgery in the consenting process.

## CONFLICT OF INTEREST STATEMENT

GA, SJR and JMGS authors are paid employees of Talita Cumi Ltd. Free From Pain is a registered trademark of Talita Cumi Ltd and is involved in providing exercise therapy for patients with neck, back and generalized musculoskeletal pain. EW has no conflict of interest to declare.

## FUNDING

This case report has been funded by Talita Cumi Ltd.

## CONSENT

The patient has provided informed consent for this case report to be published.

## GUARANTOR

GA is the Guarantor.
